# Optimized cryo-EM data-acquisition workflow by sample-thickness determination

**DOI:** 10.1107/S205979832100334X

**Published:** 2021-04-27

**Authors:** Jan Rheinberger, Gert Oostergetel, Guenter P. Resch, Cristina Paulino

**Affiliations:** a Department of Structural Biology and Membrane Enzymology at the Groningen Biomolecular Sciences and Biotechnology Institute, University of Groningen, Groningen, The Netherlands; b Nexperion e.U. – Solutions for Electron Microscopy, Vienna, Austria

**Keywords:** single-particle cryo-electron microscopy, sample thickness, automation, *Digital Micrograph*, *SerialEM*

## Abstract

Sample thickness is a known key parameter in cryo-electron microscopy (cryo-EM) and can affect the amount of high-resolution information retained in the image. Yet, common data-acquisition approaches in single-particle cryo-EM do not take it into account. Here, it is demonstrated how the sample thickness can be determined before data acquisition, allowing the identification of optimal regions and the restriction of automated data collection to images with preserved high-resolution details. This quality-over-quantity approach almost entirely eliminates the time- and storage-consuming collection of suboptimal images, which are discarded after a recorded session or during early image processing due to a lack of high-resolution information. It maximizes the data-collection efficiency and lowers the electron-microscopy time required per data set. This strategy is especially useful if the speed of data collection is restricted by the microscope hardware and software, or if microscope access time, data transfer, data storage and computational power are a bottleneck.

## Abstract 2

Sample thickness is a key parameter in single-particle cryo-electron microscopy. Determining the sample thickness before data acquisition allows the targeting of optimal areas and the maximization of the data-output quality of single-particle cryo-electron microscopy sessions. Scripts and optimized workflows for *EPU* and *SerialEM* are presented and are available as open source.

## 1. Introduction   

In single-particle cryo-electron microscopy (cryo-EM), the thin layer of vitreous ice embedding the protein or macromolecule complex of interest is a key parameter in sample preparation and optimization. Obtained by rapidly plunge-freezing grids with holey support films into liquid coolant (Adrian *et al.*, 1984 ▸), it preserves the structural integrity of the macromolecular complex of interest. The thickness of the vitreous ice layer has an impact on electron transparency and image formation and is a key criterion of image quality. Thicker layers experience a defocus gradient, which leads to a stronger dampening of higher frequencies, limiting the resolution of the final reconstruction obtained (Wu *et al.*, 2016 ▸). Conversely, an ice layer that is too thin might not be sufficient to fully embed the target protein or molecule of interest. This might lead to its denaturation at the air–water interface, or push it towards the edge of the support film hole, limiting the number of copies in the field of view (Noble *et al.*, 2018 ▸; D’Imprima *et al.*, 2019 ▸). The importance and urgency of the optimization of cryo-EM sample preparation is reflected by the development of new techniques and devices in recent years (Dandey *et al.*, 2018 ▸; Rubinstein *et al.*, 2019 ▸; Ravelli *et al.*, 2020 ▸; Tan & Rubinstein, 2020 ▸; Arnold *et al.*, 2017 ▸; Kontziampasis *et al.*, 2019 ▸; Mäeots *et al.*, 2020 ▸).

Determining the thickness of a specimen is nothing new and has been described previously (Cho *et al.*, 2013 ▸; Yan *et al.*, 2015 ▸; Rice *et al.*, 2018 ▸; Suloway *et al.*, 2005 ▸). Yet, it has been mostly calculated on high-resolution images and used to monitor sample thickness during an ongoing data collection or to select micrographs based on their sample thickness after data acquisition. In contrast, our work focuses on using sample-thickness determination at low magnification to set up automated thickness-based hole-targeting, restricting data collection to optimal regions, similar to what is available in *Leginon* (Suloway *et al.*, 2005 ▸; Cheng *et al.*, 2021 ▸). Here we have successfully integrated this approach, available as open source, in the data-acquisition software packages *SerialEM* (Mastronarde, 2005 ▸; Schorb *et al.*, 2019 ▸) and *EPU* (Thermo Fisher Scientific), and have tested it on 200 and 300 kV high-end electron microscopes, making this workflow now available for the vast majority of software packages used.

As previously described and implemented in *Leginon* (Rice *et al.*, 2018 ▸; Suloway *et al.*, 2005 ▸), two fundamentally different approaches to estimating the thickness from projection images exist, which depend on the configuration of the microscope. In the case of a microscope equipped with an energy filter, one can benefit from the thickness-dependent number of inelastic scattering events. Due to the energy loss, these electrons will be removed by the filter when operated in zero-loss mode.

The thickness *D* is proportional to the natural logarithm of the ratio between the mean intensity without (*I*) and with the slit inserted (*I*_zl_):
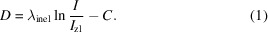



The apparent mean free path λ_inel_ acts as a scaling factor and describes the average distance that electrons travel through the sample before an inelastic scattering event occurs. The correction term *C*, which was not present in the original description (Rice *et al.*, 2018 ▸), is used by the scripts presented in this work to set the measurements in a hole over vacuum to zero.

For the aperture-limited scattering (ALS) method (2), the sample thickness can be determined by comparing the mean intensity over vacuum (*I*_0_) with the mean intensity over ice (*I*), utilizing the effect that the objective aperture removes part of the elastically scattered electrons. The number of scattered electrons is again proportional to the thickness (and content) of the sample (Angert *et al.*, 1996 ▸; Cho *et al.*, 2013 ▸; Rice *et al.*, 2018 ▸). Here, instead of the mean free path, a scaling factor termed the ALS coefficient λ_ALS_ is used, which equally depends on the acceleration voltage, the objective aperture size, the sample content (Rice *et al.*, 2018 ▸) and the imaging mode (LM versus M/SA),
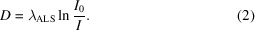



Both λ_inel_ and λ_ALS_ can be determined experimentally by tomography or comparison between both methods (Noble *et al.*, 2018 ▸; Rice *et al.*, 2018 ▸). The values are apparent and representative of the measurements in this work but not of precise physical quantities.

In this work, we present our approach for an optimized data-collection workflow by targeting only holes with optimal sample thickness. Thereby, we substantially increase the data-collection efficiency by maximizing the output quality, while minimizing the number of images collected that do not contribute to the high-resolution information and which are discarded immediately or early on during image processing. This is achieved by either combining *Digital Micrograph* (Gatan) scripts with the Filter Ice Quality histogram implemented in *EPU* (TFS) for targeting or by using scripts that combine the entire procedure in *SerialEM* (Mastronarde, 2005 ▸; Schorb *et al.*, 2019 ▸). Based on quality assessments, such as resolution estimation of the CTF fit (better than 4 Å), more than 90–95% of the data collected using this workflow provide high-resolution information.

## 2. Materials and methods   

### 2.1. Microscopy   

A Talos Arctica (TFS) equipped with a BioQuantum/K2 energy filter was operated at 200 kV in zero-loss mode (slit width 20 eV). At M910× magnification (calibrated pixel size 144.6 Å per pixel) the system was set to microprobe mode with a 50 µm C2 aperture at spot size 8, with gun lens 5 and the C2 lens at 100%, resulting in an electron flux of 12.9 e^−^ per pixel per second (10.7 counts per pixel per second). For LM690× magnification (calibrated pixel size 190.7 Å per pixel), the system was set to microprobe mode with a 50 µm C2 aperture at spot size 7, with gun lens 5 and the C2 lens at 65.0%, resulting in an electron flux of 12.9 e^−^ per pixel per second (10.7 counts per pixel per second).

A Titan Krios (TFS) equipped with a BioQuantum/K3 energy filter was operated at 300 kV in zero-loss mode (slit width 20 eV). At SA2250× magnification (calibrated pixel size 37.9 Å per pixel), the system was set to microprobe mode with a 50 µm C2 aperture at spot size 11, with gun lens 3 and an illuminated area of 40 µm, resulting in an electron flux of 22.6 e^−^ per pixel per second. For LM580× magnification (calibrated pixel size 174 Å), the system was set to microprobe mode with a 50 µm C2 aperture at spot size 8, with gun lens 3 and an illuminated area of 200 µm, resulting in an electron flux of 12.8 e^−^ per pixel per second.

To reduce the noise in the display of the thickness distribution, images were acquired with fourfold binning. They were further median-filtered in *Digital Micrograph* to reduce noise. The area of the median filter can be adjusted in the Global Tags (the default is 3 × 3 pixels).

### 2.2. Sample preparation for calibration   

Aldolase from rabbit muscle was purified as described previously (Herzik *et al.*, 2017 ▸). The size-exclusion chromatography peak fraction was concentrated to 10 mg ml^−1^, flash-frozen in liquid nitrogen and stored at −80°C. For grid preparation, the protein was thawed and first diluted to 2 mg ml^−1^ with 20 m*M* HEPES pH 7.5, 50 m*M* NaCl. The diluted solution was further mixed in a 1:10 ratio with 10 nm nano gold fiducial suspension, resulting in a final protein concentration of 1.8 mg ml^−1^. 2.8 µl of the mixture was applied onto glow-discharged (5 mA, 20 s) R1.2/1.3 holey carbon gold grids (300 mesh, Quantifoil) at 22°C and 100% humidity. The grids were immediately blotted for 2 s and plunge-frozen in liquid ethane/propane using a Vitrobot Mark IV (TFS).

### 2.3. Calibration   

To calibrate λ_inel_, the grids were loaded onto a Talos Arctica operated at 200 kV and equipped with a BioQuantum/K2 energy filter (Gatan) with slit width 20 eV. A grid square was adjusted to eucentric height and the microscope was set to the imaging conditions for the thickness measurement.

Using a default value for λ_inel_, apparent thickness values for different holes were calculated via the script. A tilt series was acquired at each of the positions at SA24000× magnification (calibrated pixel size 5.5 Å per pixel) with *TOMO* (TFS) using a tilt range from −60° to +60° with 2° increments (dose per tilt angle 0.6 e^−^ Å^2^, total dose 36 e^−^ Å^2^). *IMOD* (Kremer *et al.*, 1996 ▸; Mastronarde, 1997 ▸) was used to reconstruct tomograms using the gold fiducials as markers to align the tilt series. From the cross section of the tomograms, the actual ice thickness can be determined. Comparison of the apparent with the actual thickness value under given conditions allowed the correct λ_inel_ and λ_ALS_ values to be obtained, which are used by the *Digital Micrograph* and *SerialEM* script, respectively, when executed for the first time. For the 200 kV Talos Arctica, in the M/SA magnification range, λ_inel (200 kV, M/SA)_ is 305 nm. Comparing thickness values at the M magnification with the same grid square at the LM magnification resulted in a respective λ_inel (200 kV, LM)_ of 485 nm. For a 300 kV Titan Krios, the λ_inel (300 kV, SA)_ of 435 nm for the SA magnification range was taken from previous work (Rice *et al.*, 2018 ▸), which worked very well. The value for the LM range was determined as mentioned above and corresponded to an λ_inel (300 kV, LM)_ of 805 nm. λ_ALS_ for the LM magnification range was determined by comparison with the filter-based method (600 nm for the TFS 200 kV Talos Arctica and 1750 nm for the TFS 300 kV Titan Krios). The values of λ_inel_ and λ_ALS_ seem to be very stable and recalibration was not necessary at a later point using the same imaging condition. The fact that we could use the reported values of λ_inel_ from Rice *et al.* (2018 ▸) for the Titan Krios suggests that these values might actually be transferable and stable. However, we do not have sufficient data to ensure long-term stability and we therefore recommend checking and eventually recalibrating λ_inel_ and λ_ALS_ after any major change in microscope configuration.

## 3. Results and discussion   

### 3.1. *Digital Micrograph* and *EPU*   

**Figure 1:**
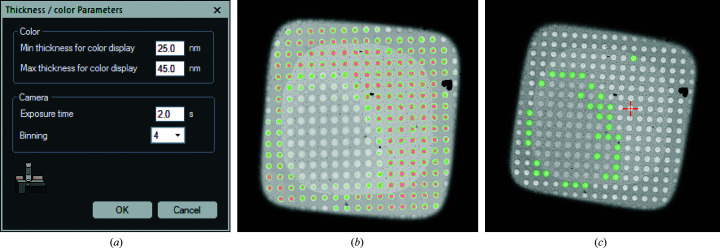
(*a*) GUI of the *Digital Micrograph* script, providing access to key parameters, for example changing the thickness range, even after script execution. (*b*) Representative color heatmap of the sample thickness obtained with the *Digital Micrograph* script. Coloring shows thickness values above (no color), within (green) and below (red) the user-defined thresholds. (*c*) Hole targeting of the same square in *EPU*, where the heat map in (*b*) was transferred by adjusting the thresholds of the *EPU* Filter Ice Quality histogram.

With the functions available through *Digital Micrograph* (version 2.2 or higher), we developed a script that allows the user to calculate and monitor the sample thickness of a grid square with a holey-carbon support film at low magnification. The respective holes are colored with a heatmap as too thick, optimal or too thin, based on a user-defined thickness range (Fig. 1 ▸ *a*). In addition, the user can determine the thickness at any point in the image.

When executed, the script will collect two images: with and without the energy-filter slit inserted. From these two images a ratio image is calculated representing *I*/*I*_zl_ (1). In the LM magnification range, where it might be difficult to align the zero-loss peak to the higher magnification, we provided the option of a magnification-dependent energy shift to compensate for the offset. The user has to individually determine and provide these shifts in the Global Tags (default 0 eV). For the ALS method, the user needs to define the mean background intensity, which is obtained by measuring the intensity over vacuum within an empty hole (*I*_0_, equation 2). This value needs to be determined only once per data-collection setup and is subsequently used to calculate the local thickness (2) within a grid-square image acquired using the same imaging settings. The switch between the filter-based and ALS methods can be activated by a parameter in the Global Tags.

The colored heatmap is obtained through a pixel-by-pixel calculation (equation 1 or 2) and the script assigns each pixel a thickness value. With respect to the user-defined threshold, it will allocate the respective color in an RGB image (too thin, red; optimal, green; too thick, no color; Fig. 1 ▸ *b*). The local measurement uses the cursor position in the image and averages the value of the ratio image within a square around these coordinates to calculate the sample thickness. The script also checks the beam intensity to prevent coincidence loss that is too high (for example below 10 counts per pixel per second for a Gatan K2 camera), thereby ensuring consistent results. While detecting the used voltage, either 200 or 300 kV, it will use the respective set of λ_inel_ and λ_ALS_ needed for the calculations. Furthermore, it can distinguish between a K2 and K3 camera (Gatan) and handle the different ways of electron counting. Everything is finished with a GUI that allows relevant parameters such as the thickness range, as well as the exposure time and binning, to be quickly changed (Fig. 1 ▸ *a*). Further parameters can be changed in the Global Tags in *Digital Micrograph*. Since *EPU* only offers dedicated presets for data collection, and microscope control via *Digital Micrograph* is limited, we wrote an additional script in *JScript* within the TFS TEM scripting environment which allows the microscope parameters to be stored and recalled to run the thickness measurement reliably.

Once the sample thickness has been determined, the gained knowledge can be transferred into *EPU*. Here, the Filter Ice Quality threshold option during hole targeting is manually adjusted to mimic the hole selection obtained in *Digital Micrograph* (Fig. 1 ▸ *c*). However, this only needs to be performed once for a representative grid square at the beginning when setting up data collection, and requires a total of only ∼5–10 min of additional user input. The same parameters will be adopted for any other regions within the same grid and no additional steps are required during data collection. Hence, with the exception that on average fewer holes per grid square are selected and imaged, data acquisition itself is not slowed compared with a conventional setup.

### 3.2. *SerialEM*   

A clear disadvantage when using *EPU* as data-collection software is that the sample thickness needs to be measured separately in *Digital Micrograph* first and subsequently transferred into *EPU*. Moreover, *EPU* is commercial software that is only available for electron microscopes from TFS. To avoid this, we were able to implement the entire workflow in the open-source acquisition software *SerialEM* (Mastronarde, 2005 ▸; Schorb *et al.*, 2019 ▸) that is compatible with multiple TEM platforms. The newly developed scripts determine the sample thickness and use the acquired values to automatically target holes within the user-defined thickness range. This setup takes advantage of recently added software functions, such as hole finder, extended by hole combiner for acquisition in multiple holes via beam-image shift. It also requires some newly introduced script commands, which are all available in *SerialEM* version 3.9 beta1 or higher. Basic parameters (mean free path length, thickness thresholds, imaging settings, correction term and averaging radius) are set as variables in the script to minimize user interaction.

**Figure 2:**
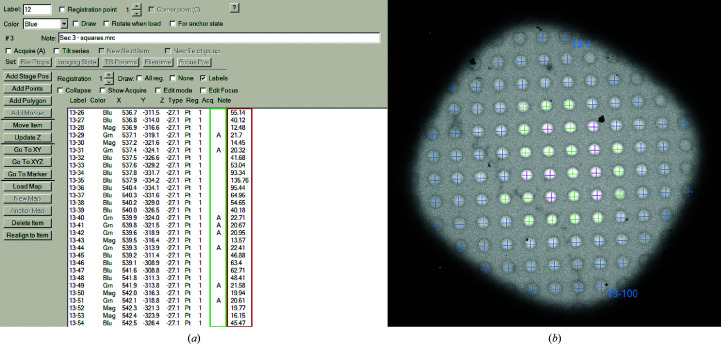
Representative output of the ice-thickness script in *SerialEM*. (*a*) Navigator window showing the calculated sample thickness in nanometres for each item (red box) and the selection for target acquisition (green box) based on the predefined thresholds 20–40 nm. (*b*) Hole positions are colored by thickness distinguished into higher (blue), within (green) and lower (magenta) with respect to the thresholds.

The script will first set the microscope to the desired imaging conditions and acquire an unfiltered and a filtered image of a selected grid square. This can be performed for multiple squares and be automatically executed via the Acquire at Items function. The newly implemented hole finder function then locates the position of each hole within the square. At each hole position the mean intensities in the unfiltered (*I*) and filtered image (*I*_zl_) are extracted within a defined radius (1), which should be carefully chosen based on the magnification and binning used. This allows the sample thickness to be calculated on a hole-to-hole basis that is stored for each item in the Navigator window of *SerialEM* as a note (Fig. 2 ▸ *a*, red box). Based on a user-defined sample thickness range, the calculated values are used to select entries for target acquisition (Fig. 2 ▸ *a*, green box) and to color positions, respectively (too thick, blue; optimal, green; too thin, magenta; Fig. 2 ▸ *b*). The acquisition of each grid square currently requires about 5 min, but the process can be automated thanks to the eucentric height routine in *SerialEM* such that the user only has to provide the locations of the squares of interest. To operate in the LM magnification range, a slightly adapted version of the script is available using the Search preset function for image acquisition. This might require an additional energy shift for the respective magnification. As this energy shift is only applied when the slit is inserted and it introduces an image shift, a correction function was implemented allowing the correct intensity values to be extracted from both the unfiltered and the filtered image. For systems without an energy filter we provide a separate script which can determine the sample thickness using the ALS method outside a *Digital Micrograph* implementation. For this, the user provides the reference intensity *I*_0_ over vacuum within an empty hole, which is divided by the intensity *I* extracted from a grid-square image (2).

The entire workflow is flexible, allowing the user to intervene at any point and make adjustments. If, for example, the initially targeted thickness range is not ideal, a second script allows the user to adjust the hole selection using the sample-thickness values stored in the Navigator note and repeat the threshold-based selection for all entries. This needs minimal user input and finishes within a minute for 5000 points.

### 3.3. Advantages and limitations   

**Figure 3:**
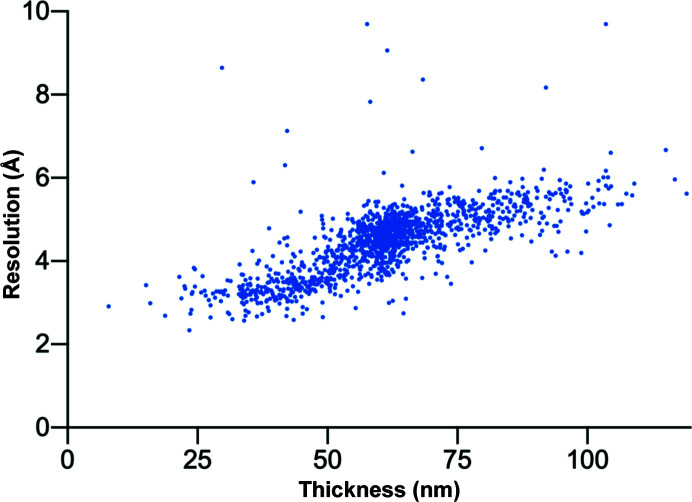
CTF resolution estimation as a function of thickness. The graph displays the resolution estimation obtained during CTF determination for over 1400 images with respect to their sample thickness, visualizing the negative effect of thicker areas.

While in our experience, based on small membrane proteins, the optimal sample thickness is in the range 20–40 nm (Fig. 3 ▸), this should be tested and optimized for each project individually. A good starting point is the expected particle size ± 5–20 nm. For this purpose, on-the-fly pre-processing software packages such as *FOCUS* (Biyani *et al.*, 2017 ▸), *Warp* (Tegunov & Cramer, 2019 ▸) or *Appion* (Lander *et al.*, 2009 ▸) are useful to provide rapid feedback. Here, calculated parameters such as the resolution of CTF estimation, particle distribution or preliminary 2D classifications can be used to assess data quality on the fly and correlate it with the measured sample thickness, allowing the user to define the optimal thickness range and adjust the targeting parameters if required.

While sample quality and thickness homogeneity can differ significantly between and within grids, the grid squares shown in Figs. 1 ▸ and 2 ▸ are, in our experience, representative. Here, conventional hole selection would have targeted the majority of all visible holes. Yet, more than 50% of the images would not meet the abovementioned quality criteria and would be discarded right after data collection or during early image processing. By contrast, our thickness-based approach restricts data collection to only a relatively small fraction of optimal holes present, with 90–95% of the data preserving high-resolution information. The remaining 5–10% of the micrographs are usually discarded due to contamination or mismatches between the position of targeting and acquisition. Compared with conventional data-acquisition workflows, this approach can thus lead to a twofold to fivefold increase in retained data.

An emerging technique to speed up data collection is the use of beam-image shift. It allows the acquisition of multiple images (for example a 3 × 3 pattern) by a combined beam and image shift instead of using stage movement (Cheng *et al.*, 2018 ▸; Wu *et al.*, 2019 ▸; Cash *et al.*, 2020 ▸). The introduced beam tilt can be corrected directly using the aberration-free image-shift (AFIS) correction in *EPU* (currently only available for the TFS Titan Krios) or the coma versus image shift calibration in *SerialEM*. Alternatively, it can be corrected later during image processing (Cash *et al.*, 2020 ▸) by using the CTF refinement functions implemented in, for example, *RELION* or *cryoSPARC* (Zivanov *et al.*, 2018 ▸; Punjani *et al.*, 2017 ▸). Ideally, one would like to combine this approach with the selection of the best thickness areas. This is possible in *SerialEM* with the newly implemented hole combiner. It only considers Navigator entries that are selected for acquisition and groups them in a user-defined pattern (for example 3 × 3). All entries within a group not marked for acquisition (*i.e.* outside the defined thickness range) will be skipped. In *EPU*, with the fast acquisition mode activated, grouping is performed automatically while considering only selected holes within a group. Both setups provide a significant boost in throughput compared with acquisition by stage movement, while keeping the benefits of optimal area selection.

Another approach that aims to minimize beam-induced movement is the use of gold-coated support films (Russo & Passmore, 2016 ▸; Naydenova *et al.*, 2020 ▸). We can confirm that our workflow also works well with commercially available UltraAuFoil grids (Quantifoil), in particular using the filter-based approach in *SerialEM*.

The thickness scripts described here have been developed and tested on TFS microscope systems, namely on a 200 kV Talos Arctica and a 300 kV Titan Krios, both equipped with BioQuantum K2/K3 energy filters. On these systems the energy filter-based method is the preferred option and is most accurate in the M or SA magnification range. This works well for a TFS Talos Arctica, as the lowest possible magnification (M910×) still includes a full square of 300-and 400-mesh grids and the majority of a 200-mesh grid square. However, for a TFS Titan Krios, the lowest SA magnification (SA2250×) covers less than a quarter of a 300-mesh grid square or about a third of a 400-mesh grid square. Alternatively, an LM magnification can be selected in combination with the filter-based approach. Although slightly less accurate, it provides a larger field of view that is able to cover an entire grid square for automatic targeting with *SerialEM* on a TFS Titan Krios. For systems without an energy filter, the ALS script available for *SerialEM* allows the use of a flexible magnification range, representing another good alternative.

We noticed that the measured reference intensity value within an empty carbon film hole was consistently higher than that measured in a fully empty area (for example a broken support film). The nature of this optical effect is not entirely clear to us, but it was more pronounced at 200 kV compared with 300 kV. Since we assume that holes with a vitreous ice layer will also experience this effect, the reference intensity for the ALS method has to be obtained within an empty hole instead of in an area with no surrounding support film. Furthermore, when measuring empty holes with the filter-based method, the calculated thickness was consistently higher than expected. This led to the introduction of the term *C* in (1), which corrects for this offset and brings the measurement back to zero as it should be. At 200 kV this value is around 4 nm for M and SA magnifications and around 35 nm in the LM range. Notably, this term appears to be unaffected by the defocus setting. For the TFS 300 kV Titan Krios, we estimate a value close to zero (1 nm) for both the M and SA magnification ranges and of about 65 nm at the described LM settings. The values for LM or M/SA can be separately specified and adjusted by the user in the Global Tags in *Digital Micrograph*.

## 4. Summary   

The possibility of specifically targeting only the best areas in a cryo-EM sample allows the optimization of the data-collection workflow. Sample thickness is a key player in cryo-EM data quality, whereby ice that is too thick lowers the amount of high-resolution information retained and ice that is too thin can reduce the number of usable particles in the field of view or affect the structural integrity of the macromolecule of interest. Our open-source scripts allow the user to calculate the thickness in a holey carbon film and set up automated data collection targeting only holes within a predefined optimal thickness range. This quality-over-quantity approach allows us to restrict imaging only to regions that will provide high-resolution information and thereby avoid the collection of suboptimal images which would be discarded right after data collection or during early image processing. We were able to successfully implement it in commonly used data-acquisition packages and make it compatible with the majority of commercially available TEMs via the open-source *SerialEM*.

While only requiring minimal additional user input, this approach maximizes the data-collection efficiency and lowers the electron microscopy time required per data set. It is particularly useful if the speed of data collection is restricted by the microscope hardware and software, or if access time to high-end microscopes, data transfer, data storage and computational power are a bottleneck. For the TFS Talos Arctica, which offers a lower acquisition rate when compared with a TFS Titan Krios (due to higher intrinsic stage drift, which requires longer waiting times, and due to the lack of a third condenser lens, which requires large beam sizes to maintain parallel illumination and does not allow multiple data acquisitions per hole), this new workflow has been demonstrated to be crucial. The approach has been routinely used in all our projects, proving its versatility and efficiency experimentally (Garaeva *et al.*, 2018 ▸, 2019 ▸; Stock *et al.*, 2018 ▸; Alvadia *et al.*, 2019 ▸; Kalienkova *et al.*, 2019 ▸; Arkhipova *et al.*, 2020 ▸; Sikkema *et al.*, 2020 ▸; Lam *et al.*, 2021 ▸).

## 5. Data availability   

The *Digital Micrograph* script can be found in the FELMI–ZFE DM-Script Database (https://www.felmi-zfe.at/dm_script/measure-thickness-in-eftem-2/). The script for setting the imaging conditions written in JScript is available on github (https://github.com/jrheinberger/SetThicknessMeasurementConditions). All *SerialEM* scripts have been deposited in the SerialEM Script Repository (https://serialemscripts.nexperion.net/script/63 and related scripts).

Further details regarding the installation and execution of the *Digital Micrograph* script can be found in the supporting information.

## Supplementary Material

Sample-thickness determination manual. DOI: 10.1107/S205979832100334X/qv5001sup1.pdf
